# Retrospective comprehensive analysis of regional lymph node recurrence in breast cancer patients (REASON study)

**DOI:** 10.1007/s00432-025-06235-5

**Published:** 2025-05-29

**Authors:** Aikaterini Liapi, Veronica Aedo-Lopez, Wendy Jeanneret-Sozzi, Athina Stravodimou, John O. Prior, Marie Nicod Lalonde, Assia Ifticene Treboux, Loic Lelievre, Lana Kandalaft, Laetitia Rossier, Audrey Goupil, Marzio Bergomi, Jean-Paul Rivals, Jean-Philippe Brouland, Elsa Curtit, Jean-Yves Meuwly, Khalil Zaman

**Affiliations:** 1 Department of Medical Oncology, Department of Oncology, Lausanne University Hospital CHUV, Rue de Bugnon 46, 1003 Lausanne, Switzerland; 2 Deprtment of Radiotherapy, Lausanne University Hospital CHUV, Lausanne, Switzerland; 3 Department of Nuclear Medicine and Molecular Imaging, Lausanne University Hospital CHUV, Lausanne, Switzerland; 4 Department of Gynecology, Lausanne University Hospital CHUV, Lausanne, Switzerland; 5 Department of Oncology, Center of Experimental Therapeutics, Lausanne University Hospital CHUV, Lausanne, Switzerland; 6 Institute of Pathology, Lausanne University Hospital CHUV, Lausanne, Switzerland; 7 Department of Oncology, University Hospital Besancon, Besancon, France; 8 Department of Radiology, Lausanne University Hospital CHUV, Lausanne, Switzerland

**Keywords:** Locoregional recurrence, Recurrent breast cancer, Lymph node, Early-stage breast cancer

## Abstract

**Background:**

Randomized trials have progressively enabled the de-escalation of axillary surgery in breast cancer (BC) patients, reducing adverse events without compromising survival. Despite a not negligible rate of residual disease in the axilla after sentinel lymph node (SLN) procedure, the risk of regional lymph node recurrence (RLNR) is very low, due probably to multimodal adjuvant treatments. The characteristics of the small number of patients with RLNR remain poorly characterized and warrant further investigation, especially given their poor prognosis and the current context of ongoing studies exploring further de-escalation of axillary surgery.

**Methods:**

In this retrospective and single institution study, we analyzed thoroughly a cohort of patients who experienced RLNR as first event between 2009 and 2020. MammaPrint and BluePrint analysis (MB) was performed in available primary invasive cancer tissues.

**Results:**

Forty patients, median age of 52, were analyzed. Disease-free interval was 8.7 years. Most of the patients (65%) had no special type BC. Majority (73%) had hormone receptor positive-HER2 negative (HR + /HER2−) BC, 13% triple negative (TNBC), 6% HER2 + , 8% ductal carcinoma in situ and 3% unknown. The median size of the primary tumor was 1.8 cm (range 0.3–7.0) and 57% had no initial LN involvement. Forty five percent had primary SLN procedure and 53% axillary LN dissection (ALND) of the patients received neo-/adjuvant chemotherapy, 63% endocrine therapy and 68% radiotherapy (50% only in breast). Sixty three percent had only RLNR and 38% had concomitant distant metastases. Among irradiated patients, 63% had some relapse in the radiation field. The MB analysis classified 70% of the analyzed cancers as low-risk luminal A (82% in HR + /HER2−), 15% high-risk luminal B, 10% high-risk basal type, and 5% high-risk HER2 type.

**Conclusion:**

Our study confirms that patients treated with SLN do not show a higher risk of LRNR compared to ALND. LRNR is often diagnosed incidentally. Younger age, residual disease post-NAC, no regional radiation, stage II, and initial LN involvement were more represented, as well as patients with endocrine sensitive disease classified as low-risk luminal A by MB. Ongoing trials, including SOUND, INSEMA, and BOOG 2013-08, are further exploring axillary surgery de-escalation.

## Introduction

Breast cancer (BC) is the most common malignancy and the leading cause of cancer-related death among women in Western countries. The majority of breast cancers are diagnosed as non-metastatic, and treatments aim for cure. Standard treatments include surgery to excise the primary tumor and assess the locoregional disease extension, complemented by (neo-)adjuvant treatments, such as radiotherapy (RT), chemotherapy, endocrine therapy and targeted therapies, based on the specific BC biology (Curigliano et al. 2023). Over the last decades, these additional treatments have significantly decreased the risk of disease recurrence. Nevertheless, recurrence may still occur as distant metastases, locally in the ipsilateral or contralateral breast, or in regional lymph nodes (LNs), even following appropriate surgical intervention. Regional LN recurrence involves the ipsilateral LNs, including axillary, supraclavicular, infraclavicular and internal mammary chains.

Axillary surgery can be therapeutic in cases of macroscopic LN involvement but primarily serves as a staging procedure in patients without clinical LN enlargement. However, its role in guiding adjuvant systemic treatment decisions has been decreasing (Weber et al. 2023). Recent trends show a continuous de-escalation of axillary surgery to reduce related morbidities, such as lymphedema, neuropathy, pain and mobility impairment. Various randomized trials have demonstrated that the excision of the sentinel lymph nodes (SLNs) alone was adequate for staging in patients without LN infiltration (Boughey et al. 2023; Donker et al. 2014). The false-negative rate (FNR) of the SLN procedure is less than 10% and axillary recurrence is rare (Van der Ploeg et al. 2008). The IBCSG 23/01 trial showed that axillary lymph node dissection (ALND) is unnecessary in patients with micrometastatic SLN involvement (Boughey et al. 2023). Furthermore, the ACOSOG Z0011 trial revealed that even in the case of one or two positive SLNs, ALND can be avoided in patients without initial clinical LN enlargement (Boughey et al. 2023). The AMAROS trial demonstrated that in patients with cT1-2 BC and positive SLN, regional irradiation can replace ALND with a lower risk of lymphedema (Donker et al. 2014). More recently, the SENOMAC trial confirmed the results of ACOSOG Z0011 in a larger study (Boniface et al. 2024).

In all these trials, the risk of regional LN recurrence was low in the absence of ALND: 1% in IBCSG 23/01 (Boughey et al. 2023), 1,5% in ACOSOG Z0011 (Giuliano et al. 2016), 0.4% in SENOMAC (Boniface et al. 2024) and 1.82% in AMAROS (Donker et al. 2014). These rates contrast with the relatively high rates of residual disease in the axilla among patients in the ALND arms of these trials (13%, 27.3%, 34.5% and 33% in IBCSG 23/01, ACOSOG Z0011, SENOMAC and AMAROS, respectively). The low LN recurrence rates despite residual disease probably reflect the additional effect of adjuvant RT and systemic therapies. Results from these trials have changed the standard of care, sparing many patients from unnecessary extensive surgery.

Nevertheless, given the high incidence of breast cancer, even a small fraction of patients with regional LN recurrence represents a substantial number of women. In addition, regional LN recurrence is strongly associated with an increased risk of distant relapse and mortality (Montagna et al. 2012; Wapnir et al. 2006).

The characteristics of patients experiencing regional LN recurrence are poorly understood. Therefore, we aimed to investigate a cohort of BC patients with regional LN recurrence, analyzing clinical, pathological and therapeutic factors to better understand the risk factors for regional recurrence. This is particularly relevant as the de-escalation of axillary surgery continues with ongoing trials such as SAKK 23/16 TAXIS (NCT03513614) and Alliance A011202 (NCT01901094) for clinically node-positive BC, and SOUND (NCT02167490), INSEMA (NCT02466737) and BOOG 13-08 (NCT02271828) for node-negative BC. In addition, the SLN procedure after neoadjuvant chemotherapy is increasingly used in patients initially diagnosed with LN-positive disease (Boughey et al. 2023).

## Materials and methods

This retrospective study enrolled patients aged 18 years and older with regional LN recurrence of BC, as a first event, with or without distant metastasis, between 2009 and 2020. All patients had undergone surgery of the primary BC with curative intent. Patients were identified from the medical registry of the Breast Center of Lausanne University Hospital (CHUV), Lausanne, Switzerland. The study was approved by the local ethical committee of the Cantonal Commission for Ethics in Research on Human Beings (CER-VD) (No. 2020-00131). According to the Swiss regulation, written consent was obtained from all available participants. Some patients had provided institutional general consent, permitting further use of their samples and data. Patients who had not signed general consent provided a specific individual informed consent before their inclusion in the trial. For deceased patients, material use was authorized by the ethical committee according to Art. 34 HRA (Federal Act on Research involving Human Beings 2011), allowing research projects without informed consent under specific conditions. There were no cases of revoked consent. This study was conducted in accordance with the Declaration of Helsinki (World Medical Association 2024).

**Fig. 1:**
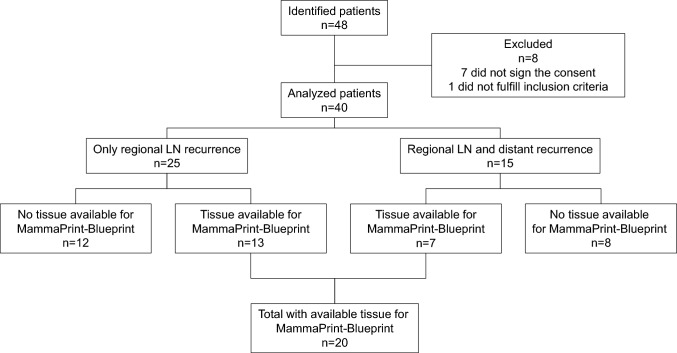
CONSORT diagram

This observational, descriptive and hypothesis-generating study had no prespecified sample size. The study instead aimed for an in-depth analysis of a small number of patients, with the size determined by the number of patients fulfilling the selection criteria. Forty patients were ultimately included and analyzed out of 48 patients assessed for eligibility (Fig. 1).

We collected demographic and clinical data, including age at primary diagnosis and recurrence, weight, time to recurrence, mode of LN recurrence detection (clinical versus radiological) and BReast CAncer gene (BRCA) status, when available.

We further reviewed the available radiological exams, including mammogram, breast ultrasound, MRI, PET and CT scans, performed at primary diagnosis and at recurrence. We analyzed the location of the LN recurrence relative to the primary cancer location, the initially involved LN, if present, and the field of the adjuvant RT administered.

Concerning the primary surgery history, we identified the type of initial surgery: breast-conserving surgery (BCS) versus mastectomy, SLN procedure alone, SLN procedure followed by ALND or ALND alone. We reviewed the levels of dissection (I, II or III per Berg’s classification) in case of ALND, the timing of SLN procedure relative to surgery (simultaneous or sequential) and the location of the SLN relative to the LN recurrence site. Information was collected from surgical protocols and radiological files.

The following histopathological data were collected for each primary tumor: tumor size (T) in mm, presentation type (unifocal, multifocal or multicentric), regional LN involvement (N), positive/negative LN ratio, presence of concomitant ductal carcinoma in situ (DCIS), histologic subtype, expression of estrogen receptors (ER), progesterone receptors (PgR), human epidermal growth factor receptor 2 (HER2) and Ki67, lymphovascular invasion, and results of genomic tests, if available. For LNs, the presence or absence of extracapsular extension was reported. In cases of SLN procedure alone, we analyzed the number of SLNs removed versus satellite LNs. The biology of the primary tumor was compared to that of the recurrent cancer.

For patients with available invasive primary BC tissue samples were collected. MammaPrint and BluePrint analyses (Agendia, Amsterdam, The Netherlands) were performed on these primary cancer samples independently of BC subtype defined by immunohistochemistry (IHC). MammaPrint is a 70-gene signature that stratifies early-stage BC patients by risk of distant relapse (low versus high), while BluePrint, an 80-gene molecular subtyping test for early-stage BC, classifies the disease into basal-type, luminal-type and HER2-type. Adjuvant systemic treatments administered before regional LN recurrence were documented. RT files were reviewed for radiation fields, total dose and radiation field location compared with the regional LN recurrence.

Descriptive statistics were presented as absolute frequency (number) and relative frequency (percentage) for categorical variables. Normally distributed continuous variables were reported as means and non-normally distributed continuous variables were presented as medians.

## Results

A total of 40 female patients with regional LN recurrence of BC, with or without distant metastases, were eligible and included in the study. They represent 1.7% of the 2357 patients with early-stage BC treated at the center between 2009 and 2020. Seventeen patients had genetic test; three had *BRCA2* and one had *BRCA1* pathogenic variant. The median disease-free interval (time between the primary cancer diagnosis and the regional relapse) was 8.7 years (range: 0.6–34.9). The median follow-up after the regional recurrence was 3.5 years.

The characteristics of the study patients are detailed in Table 1. most common histologic type was No Special Type (NST) carcinoma. At initial diagnosis, half of the patients had stage II disease. The median size of the primary BC was 18 mm. LN involvement at initial diagnosis was present in 17 patients (43%). Hormone receptor (HR)-positive, HER2-negative BC was the predominant subtype.

**Table 1:** Disease characteristics at baseline

Variable	Category		All,* n=40 *(%)	SLN, *n=18* (%)	ALND *n=21* (%)
Primary cancer					
Stage (all)	I	14 (35)		7 (17.5)	7 (17.5)
	II	20 (50)		8 (20)	12 (30)
	III	2 (5)		1 (2.5)	1 (2.5)
	0	3 (7.5)*		2 (5)	0 (0)
	Unknown	1 (2.5)		0 (0)	1 (2.5)
Stage among HR+/HER2− only (*n=29*)	IA	13 (45)		6 (20.7)	7 (24.1)
	IIA	10 (34)		4 (13.8)	6 (20.6)
	IIB	4 (14)		1 (3.4)	3 (10.3)
	IIIA	2 (7)		1 (3.4)	1 (3.4)
Node involvement	Yes	17 (42.5)		5 (12.5)	12 (30)
	No	23 (57.5)*		13 (32.5)	9 (22.5)
Number of LN ablation in patients with SLN alone	1	7		7	
	2	4		4	
	3	5		5	
	Unknown	2		2	
Lymphovascular invasion	Yes	11 (27.5)		4 (10)	7 (17.5)
	No	21 (52.2)*		13 (32.5)	7 (17.5)
	Unknown	8 (20.0)		1 (2.5)	7 (17.5)
Histologic type	Invasive NST	26 (65)		11 (27.5)	15 (37.5)
	Invasive lobular	7 (17.5)		3 (7.5)	4 (10)
	DCIS	3 (7.5)*		2 (5)	0 (0)
	Invasive NST and lobular	1 (2.5)		1 (2.5)	0 (0)
	Invasive micropapillary	1 (2.5)		0 (0)	1 (2.5)
	Invasive apocrine	1 (2.5)		1 (2.5)	0 (0)
	Invasive mucinous	1 (2.5)		0 (0)	1 (2.5)
Subtypes	HR+/HER2−	29 (72.5)		12 (30)	17 (42.5)
	HR+/HER2+	1 (2.5)		1 (2.5)	0 (0)
	HR−/HER2+	1 (2.5)		1 (2.5)	0 (0)
	HR−/HER2−	5 (12.5)		2 (5)	3 (7.5)
	DCIS	3 (7.5)*		2 (5)	0 (0)
	Unknown	1 (2.5)		1 (2.5)	0 (0)

*SLN* sentinel lymph node, *ALND* axillary lymph node dissection, *DCIS* ductal carcinoma in situ, *HER2* human epidermal growth factor receptor 2, *HR* hormone receptor, *NST* no special type

^*^: One patient with initial DCIS

Surgical and neo-/adjuvant treatments data, presented in Table 2, showed that breast-conserving surgery was slightly more common than mastectomy. Nearly all patients had axillary surgery, either SLN procedure alone, ALND or SLN procedure followed by ALND.

**Table 2:** Treatments of primary cancer

Variable	Category	Cases, *n* = *40* (%)	SLN *n* = *18* (%)	ALND *n* = *21* (%)
Surgical treatment for primary cancer				
Primary surgery	BCS	23* (58)	12 (30)	10 (25)
	Mastectomy	17 (43)	6 (15)	11 (27.5)
Systemic treatment of the primary cancer				
NAC (n = 5)	Anthracycline	1 (2.5)	1 (2.5)	0 (0)
	Anthracycline and taxane	3 (7.5)	1 (2.5)	2 (5)
	Taxane and platinum	1 (2.5)	1 (2.5)	0 (0)
Adjuvant chemotherapy (n = 15)	Anthracycline	6 (15)	2 (5)	4 (10)
	Anthracycline and taxane	6 (15)	2 (5)	4 (10)
	Taxane based only	1 (2.5)	1 (2.5)	0 (0)
	Other	2 (5)	1 (2.5)	1 (2.5)
Anti-HER2 treatment (n = 2)	Trastuzumab	1 (2.5)	1 (2.5)	0 (0)
	Trastuzumab and pertuzumab	1 (2.5)	1 (2.5)	0 (0)
Endocrine therapy (n = 25)	Tamoxifen	10 (25)	6 (15)	4 (10)
	Aromatase inhibitor	7 (17.5)	2 (5)	5 (12.5)
	Tamoxifen and aromatase inhibitor	8 (20)	3 (7.5)	5 (12.5)
Radiation therapy (n = 27)	Breast/chest only	22* (55)	10 (25)	11 (27.5)
	Locoregional	5 (12.5)	3 (7.5)	2 (5)

*ALND* axillary lymph node dissection, *BCS* breast-conserving surgery, *HER2* human epidermal growth factor receptor 2, *LN* lymph node, *SLN* sentinel lymph node

^*^: One patient with initial DCIS

Half of the patients received chemotherapy, including five patients who had neoadjuvant chemotherapy (NAC); all presented with residual disease at surgery. Patients with HER2-positive disease received adjuvant trastuzumab. Adjuvant endocrine therapy was administered to most of the patients with endocrine-sensitive disease. In addition, two-thirds of the patients received adjuvant RT either whole breast or less frequently locoregional irradiation. More treatment details are provided in Table 2.

**Fig. 2:**
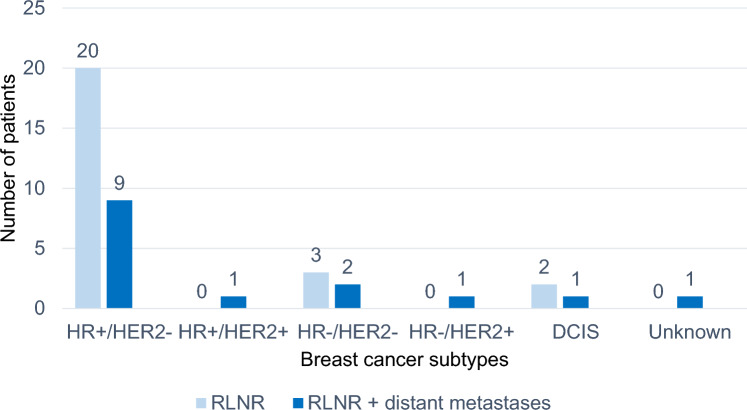
Primary breast cancer subtypes and extend of recurrence. *DCIS* ductal carcinoma in situ, *HR* hormone receptor, *HER2* human epidermal growth factor receptor 2, *RLNR* regional lymph node recurrence

**Fig. 3:**
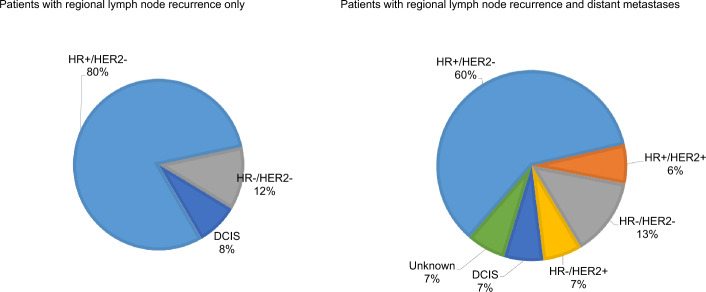
Biology of the primary cancer according to the sites of recurrence. *DCIS* ductal carcinoma in situ, *HR* hormone receptor, *HER2* human epidermal growth factor receptor 2

Regional LN recurrence was detected as much by symptomatic presentation or clinical examination as by imaging. At the moment of recurrence diagnosis, 25 patients (63%) had regional recurrence only while 15 patients (38%) presented with both regional recurrence and simultaneous distant metastases. Recurrent disease was mostly HR-positive, HER2-negative. Detailed information is provided in Table 3. Among patients with regional LN recurrence and simultaneous distant metastases, mosthad HR-positive, HER2-negative primary disease. Figures 2, 3 present recurrence sites by primary cancer biology.

**Table 3:** Disease characteristics at recurrence

Disease characteristics at recurrence		
Variable	Category	Cases,* n* (%)
Subtypes	HR +/HER2−	26 (65.0)
	HR +/HER2+	3 (7.5)
	HR−/HER2+	2 (5.0)
	HR−/HER2−	8 (20.0)
	Unknown	1 (2.5)

*HR* hormone receptor, *HER2* human epidermal growth factor receptor 2

In patients with available primary invasive BC tissue samples, MammaPrint and BluePrint genomic testing was performed. Tumor tissue sample was available for genomic testing in 13 of the 25 with LN-only recurrence and 7 of the 15 patients with LN and simultaneous distant metastases (Fig. 1). Out of the 20 samples analyzed, 70% were classified as low-risk/luminal A. Among those with LN-only recurrence, 85% were classified as low-risk/luminal A (Table 4). BC subtypes were concordant between IHC and BluePrint.

**Table 4:** Results from the MammaPrint and BluePrint analyses of primary cancer samples

MammaPrint and BluePrint classification in primary cancer	Sites of recurrence	
	Regional lymph node recurrence (n = 13)	Regional lymph node recurrence and distant recurrence (n = 7)
Low-risk/luminal A (n = 14)	11	3
High-risk/luminal B (n = 3)	1	2
High-risk/basal type (n = 2)	1	1
High-risk/HER2 type (n = 1)	0	1

*HER2* human epidermal growth factor receptor 2

**Fig. 4:**
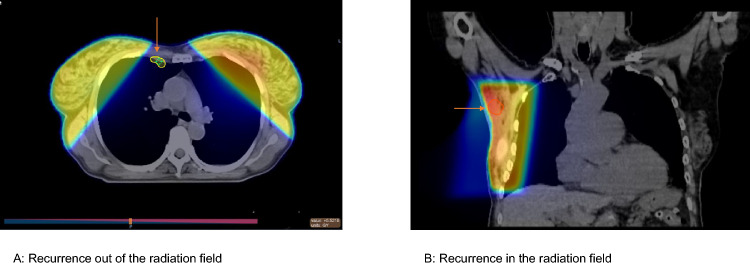
Examples of adjuvant radiation field and the location of regional recurrence. **A** recurrence out of the radiation field, **B** recurrence in the radiation field

Correlating the individual adjuvant radiation fields with regional LN recurrence sites revealed that the recurrence events occurred often fully or partially within the radiation field. We present two examples of recurrence outside the radiation field and within the radiation field in Fig. 4.

## Discussion

The aim of our study was to improve our understanding of the characteristics of patients with regional LN recurrence, which is relatively rare, and to investigate clinical and pathological factors that may contribute to the risk of regional LN recurrence in BC. With a median age of 52 years at diagnosis of their primary cancer, our patients were younger compared with the general population of BC patients both in Switzerland, as reported by the Swiss National Institute for Cancer Epidemiology and Registration (NICER) (median, 63 years) (Krebsbulletin 2015), and in the United States (median, 60 years) (Stapleton et al. 2018).

Regional LN recurrence was identified through clinical examination in 50% of patients and through imaging in 45%, aligning with data from a recent prospective Irish study showing 46% diagnosed clinically and 54% radiologically (Horan et al. 2023). In patients who underwent BCS, current guidelines generally recommend mammogram as the follow-up modality, although this approach has limited efficacy in detecting LN abnormalities (Loibl et al. 2023; Khatcheressian et al. 2013). Recent ESMO guidelines propose combining mammography with ultrasound, which can potentially increase the likelihood of radiological detection of LN recurrence. However, some studies question the value of routine LN recurrence screening due to low detection rate of recurrence events (Snider et al. 2006; Kwon et al. 2018). In addition, imaging after mastectomy is not recommended due to the very low probability of detecting recurrent disease (Smith et al. 2022). Regional LN recurrence is relatively rare and therefore often detected incidentally during radiological or clinical assessment rather than as a part of a planned follow-up strategy. The value of clinical exams seems also relatively limited compared with patient self-diagnosis for detecting LN recurrence (Horan et al. 2023; Tun et al. 2022).

In our study, the histological distribution was typical, with two-thirds of the patients classified as invasive NST and around 15% as invasive lobular carcinoma (Rakha et al. 2023). Most patients initially presented with stage I or II disease, like the staging observed in Northern and Western Europe (around 80%); however, stage II was more common in our cohort (50% of patients with regional LN recurrence) (Benitez Fuentes et al. 2023). Three women with initial DCIS also experienced LN recurrence, probably due to an occult invasive component in the primary DCIS. Notably, very low rates of DCIS with LN involvement have been reported (Davey et al. 2022; Shin et al. 2021).

LN involvement was present in 42% of patients at primary surgery, which appears somewhat higher than expected in the general BC population at the first diagnosis. In fact, the most recent US Cancer Statistics report a 25% rate of regional involvement (Siegel et al. 2024). It is well established that initial LN involvement increases the risk of locoregional recurrence. Concerning axillary surgery, ALND and SLN procedure were equally represented, indicating that the SLN procedure does not appear to confer an increased risk of LN recurrence compared with ALND.

Adjuvant RT was administered to 68% of our patients, with 55% receiving whole-breast irradiation and 13% receiving locoregional irradiation. When comparing RT fields with the location of regional LN recurrence, we found that 63% of recurrences occurred partially or fully within the radiation field, while 37% occurred outside it. Due to its tangential fields, whole-breast irradiation is known to decrease the likelihood of regional LN recurrence, as demonstrated in the ELIOT trial, where 15-year LN relapse rates were 0% in the whole-breast irradiation arm compared with 1% intraoperative partial breast radiation arm (p = 0.012) (Orecchia et al. 2021). Nevertheless, breast irradiation alone may not be as effective as dedicated regional irradiation in decreasing the risk of regional LN recurrence (Taylor et al. 2023). In a recent study including 2,162 patients from the Massachusetts General Hospital, delivering < 50 Gy (45–46.8 Gy in 25–26 fractions) to the axilla was associated with a significantly higher risk of axillary recurrence (HR: 3.0; p = 0.04). The study also identified extranodal disease in axillary soft tissue as an additional risk factor for LN recurrence (Naoum et al. 2024). Supported by the AMAROS, MA.20 and EORTC 22922 trials, the increased use of adjuvant regional LN irradiation in patients with LN involvement will likely continue to reduce the risk of regional LN recurrence (Moreno et al. 2017).

Regarding BC phenotype, the great majority of our patients with regional LN recurrence had HR-positive, HER2-negative disease, while HER2-positive or TNBC subtypes were less common. In the subgroup of patients with regional LN-only recurrence, 80% had HR-positive, HER2-negative disease, which is epidemiologically the most frequent BC subtype. This predominance can be partially explained by a higher rate of initial LN involvement at diagnosis in this subpopulation, as shown in the Surveillance, Epidemiology and End Results (SEER) registry analysis (Mattes et al. 2015). Furthermore, patients with HR-positive, HER2-negative BC also tend to have a lower rate of pathological complete response after NAC compared with patients with HER2-positive or TNBC subtypes (Hemert et al. 2024). Interestingly, none of our patients with regional LN recurrence who underwent NAC achieved pathological complete response. Locoregional recurrence is a rare event in HER2-positive BC, probably due to the incorporation of trastuzumab in the (neo-)adjuvant treatment of this subtype (Lanning et al. 2015).

Most patients in our cohort were diagnosed before the routine use of the multigene assays. Only one patient, a 52-year-old woman with stage IA, ER-positive, PR-negative, HER2-negative and a Ki67 index of 20%, had an Oncotype Dx recurrence score (RS) of 15, indicating a low risk of cancer recurrence, and did not receive adjuvant chemotherapy. We retrospectively performed MammaPrint and BluePrint testing in all the patients with available tissue from primary cancers and, 70% of our patients had disease classified as low risk and luminal A. Published data have shown that patients with genomic signatures predicting a high risk of distant relapse are also at a higher risk of locoregional recurrence (Mamounas et al. 2020). For example, 5-year rates of locoregional recurrence were 1% for luminal A, 2% for luminal B, 8% for HER2-enriched and 7% for TNBC (Nguyen et al. 2008). However, these trials were mainly conducted before the introduction of adjuvant trastuzumab and thus may underestimate outcomes for patients with HER2-positive BC. In one study, the 10-year locoregional recurrence rates were 6% for MammaPrint low-risk scores and 13% for MammaPrint high-risk scores (HR: 1.73, p = 0.042) (Drukker et al. 2014). Although these genomic signatures have some predictive ability, the difference in locoregional recurrence rates between the low-risk and the high-risk groups is not substantial, suggesting a relatively low negative predictive value. Therefore, our data are consistent with published studies assessing the genomic signatures in predicting the risk of locoregional recurrence, especially considering the high prevalence of HR-positive, HER2-negative disease, which may surpass the discriminating capacity of genomic tests. An analysis of the SEER database between 2019 and 2020, which included 320,124 BC women, showed that luminal A subtype was the most frequent, accounting for 73% of cases, followed by luminal B (11%), TNBC (11%) and HER2-enriched (5%) (Acheampong et al. 2020).

The limitations of our study include the small sample size, which reflects the relative rarity of regional LN recurrence. Furthermore, recruiting consecutive patients in a retrospective monocentric study may introduce selection bias. Primary cancer tissue was not available for the entire cohort, which could bias MammaPrint and BluePrint results. The small numbers of patients with TNBC and HER2 + BC does not allow any conclusion in these subpopulations. In patients with regional LN recurrence and synchronous distant metastases, it remains unclear whether the systemic spread of the disease was secondary to the LN involvement or directly from the primary BC. Of note, a previous study analyzing patients with de novo metastatic BC showed that there was a concurrent LN involvement in 86% of cases, suggesting a potential causative link (Bitencourt et al. 2020).

The median disease-free interval in our trial was longer compared to many previously published studies. This difference may be potentially related to factors such as differences in patient characteristics, the sample size, and especially to the retrospective nature of our study, which allowed for the inclusion of recurrences over an extended period.

## Conclusion

Regional LN recurrence remains a rare event, even with the progressive de-escalation of axillary surgery. Patients treated with SLN procedure do not appear to have a higher risk or LN recurrence than those treated with ALND, supporting the safety of axillary surgery de-escalation over the last decades. Recurrence diagnosis mainly happens incidentally, without impact from routine clinical and imaging diagnostic procedures. The phenotype of cancer that relapses regionally resembles that of the broader BC population. Younger age, residual disease after NAC chemotherapy, lack of regional adjuvant irradiation, stage II disease and initial LN involvement appear more common among patients with regional LN recurrence. Large ongoing trials will provide additional insights into patients with regional LN recurrence. For example, the first results of SOUND (Gentilini et al. 2023) and INSEMA (Reimer et al. 2024) trials already support the safety of omitting the axillary surgery in low-risk patients without clinical or imaging evidence of LN involvement. BOOG 2013-08 results are still pending. In addition, the Alliance A011202 trial is comparing ALND to regional nodal irradiation in patients with cN1 who convert to ycN0 but remain pN+ at surgery. The SAKK 23/16 TAXIS trial is assessing tailored axillary surgery as an alternative to ALND in patients with positive axillary LN and examines the role of MammaPrint and BluePrint in guiding treatment decisions.

## Data Availability

No datasets were generated or analysed during the current study.
